# Citrinin-Induced Cellular Damage: Insights from SH-SY5Y Cell Line Studies

**DOI:** 10.3390/foods14030356

**Published:** 2025-01-22

**Authors:** Francisco J. Martí-Quijal, Felipe Franco-Campos, Francisco J. Barba, María-José Ruiz

**Affiliations:** 1 Research Group in Innovative Technologies for Sustainable Food (ALISOST), Nutrition, Food Science and Toxicology Department, Faculty of Pharmacy, Universitat de València, Avda. Vicent Andrés Estellés, s/n, 46100 Burjassot, València, Spain; francisco.j.marti@uv.es (F.J.M.-Q.); francisco.barba@uv.es (F.J.B.); 2 Research Group in Alternative Methods for Determining Toxics Effects and Risk Assessment of Contaminants and Mixtures (RiskTox), Laboratory of Food Chemistry and Toxicology, Faculty of Pharmacy and Food Science, University of Valencia, Av. Vicent Andrés Estellés, s/n, 46100 Burjassot, València, Spain; m.jose.ruiz@uv.es

**Keywords:** citrinin, cell cycle, SH-SY5Y, ROS, mitochondrial membrane potential

## Abstract

Citrinin (CIT), a mycotoxin commonly found in cereals, is produced by fungi from the *Aspergillus*, *Penicillium*, and *Monascus* genera. While its nephrotoxic effects are well studied, its impact on neurons is less understood. This study investigates CIT-induced toxicity in human neuroblastoma cells (SH-SY5Y). The IC_50_ values for cells treated with CIT were 77.1 μM at 24 h and 74.7 μM at 48 h using MTT assay, and 101.0 μM at 24 h and 54.7 μM at 48 h using neutral red assay. CIT exposure caused G2/M phase arrest, with cells in this phase increasing from 11.83% (control) to 33.10% at 50 μM CIT. At 50 μM, the percentage of cells in the S phase also increased, which may suggest that cellular stress pathways were activated. Moreover, an increase in late apoptosis process was noted in cells exposed to CIT for 24 h, particularly at the highest concentrations (38.75 and 50 µM). Western blot analysis confirmed a rapid change in the anti-apoptotic protein Bcl-2, but no significant changes in Bax. In conclusion, CIT induces apoptosis and cell cycle arrest in SH-SY5Y cells. However, further transcriptomic studies in specific proteins involved in different pathways described in this work are needed to gain a comprehensive understanding of the specific mechanisms underlying CIT’s toxicity in SH-SY5Y cells.

## 1. Introduction

Mycotoxins, toxic metabolites produced by certain fungi, represent a critical issue in food safety due to their prevalent occurrence in agricultural products and processed foods, including cereals, fruits, nuts, and fermented products [1,2]. Among these mycotoxins, citrinin (CIT), primarily produced by fungi from the genera *Aspergillus*, *Penicillium*, and *Monascus*, has raised growing concern owing to its nephrotoxic and hepatotoxic effects [3,4]. The presence of CIT in food matrices presents considerable challenges for the food industry and regulatory agencies, as its presence compromises food safety and increases risks to human health. According to the last report of the European Food Safety Authority (EFSA), there is insufficient scientific evidence on this mycotoxin, which hinders proper risk assessment. This report and the interest in the effects of mycotoxins on biological systems has underscored their potential role in the development of diseases through acute and chronic exposure. Consequently, there is a pressing need for more toxicological data concerning this mycotoxin to enhance the accuracy of risk assessments and to elucidate its toxic effects [5]. Among various cell lines, the human SH-SY5Y cell line, known for its ability to replicate key biological pathways, serves as an essential in vitro model for exploring this cytotoxic damage and for understanding the mechanisms induced by mycotoxins [6,7]. Additionally, Regulation (EC) No. 1881/2006 by the European Commission, which sets the maximum contaminant levels in food and animal feed to protect public health, animal welfare, and agricultural efficiency, does not establish a specific threshold for CIT in food because of inadequate data regarding its occurrence [8]. Currently, the regulation only addresses CIT levels in food supplements. Nevertheless, there is ongoing focus and effort to improve the risk assessment of this mycotoxin.

Citrinin was detected in a wide range of foods and food supplements, with concentrations varying between 0.10 and 44,240 µg/kg [9]. In 2012, the European Food Safety Authority (EFSA) established a tolerable daily intake (TDI) of 0.2 μg/kg body weight per day as the threshold for safe nephrotoxicity levels [5]. According to research by Ali and Degen [10], human exposure levels to citrinin varied across different population groups, ranging from 1.8 ± 1.1% to as high as 103.3 ± 244.1% of the TDI.

Regarding the toxicological aspects, CIT exhibits antibacterial, antifungal, and antiprotozoal activities. However, it is also recognized as an hepatorenal toxin in several species. Studies conducted in vitro have demonstrated that CIT negatively impacts renal mitochondrial function and the synthesis of macromolecules, leading to the death of the cell [11,12]. Furthermore, in vitro studies in renal proximal tubule epithelial cells (hRPTECs) suggest that CIT may induce chromosomal instability, indicating a potential for carcinogenicity [12].

Different investigations provided evidence that exposure to CIT can lead to alterations in cell cycle progression, disrupting the tightly regulated processes that govern cellular division [13,14]. This disruption of the cell cycle can have relevant implications for cellular proliferation and function.

Moreover, the mycotoxin CIT was also found to exert significant influence on cellular apoptosis and necrosis processes. CIT was shown to trigger apoptosis by activating different cellular signaling pathways, including the mitochondrial pathway, endoplasmic reticulum stress, and caspase activation [13,15,16]. Additionally, CIT was associated with the disruption of mitochondrial membrane potential, leading to mitochondrial dysfunction and subsequent apoptotic cell death [17,18]. Moreover, CIT exposure was linked to induced damage to cellular structures and organelles [19].

Recent studies underscore the importance of investigating the toxicological effects of citrinin (CIT) in cellular models, particularly regarding its influence on essential biological processes [20,21]. Our previous research highlighted the value of using the human neuroblastoma SH-SY5Y cell line to evaluate and explore the potential of natural extracts from marine by-products, such as fishery residues and microalgae like Spirulina, to counteract CIT-induced effects [22]. Building on these findings, the present study aims to deepen our understanding of CIT’s cytotoxicity by focusing on specific toxicity mechanisms. Using the human neuroblastoma cell line SH-SY5Y as an established in vitro model, we aim to explore the cellular pathways disrupted by CIT exposure.

## 2. Materials and Methods

### 2.1. Chemicals

The reagents used for cell culture and assays were supplied by Sigma Chemical Co. (St. Louis, MO, USA). DMSO was provided by Fisher Scientific (Geel, Belgium). Ethanol (CAS: 64-17-5) and NaCl (7647-14-5) were purchased from Merck (Darmstadt, Germany), while CaCl_2_ (CAS: 10043-52-4) was obtained from Scharlau Chemie S.A. (Barcelona, Spain). Materials and reagents for Western blot analysis were procured from Bio-Rad Laboratories (Hercules, CA, USA). Annexin V-FITC conjugate was provided by Invitrogen (Thermo Fisher Scientific, Waltham, MA, USA). The mycotoxin standard, CIT (MW: 250.25 g/mol; CAS: 518-75-2), was obtained from Sigma-Aldrich (St. Louis, MO, USA), with stock solutions dissolved in DMSO and stored in the freezer at −20 °C.

### 2.2. Cell Cultures and Treatment

SH-SY5Y human neuroblastoma cells (ATCC CRL-2266), sourced from ATCC (American Type Culture Collection), were incubated in DMEM Ham’s-F12 medium supplemented with streptomycin (100 mg/mL), fetal bovine serum (FBS) (10% (*v*/*v*)), and penicillin (100 U/mL). The cells were cultured under normal conditions at 37 °C, pH 7.4, 95% air humidity, and 5% CO_2_ [23]. CIT solutions were formulated to maintain a final DMSO concentration of 1% (*v*/*v*) or less. Control groups with equivalent solvent amounts were included in all experiments. The absence of mycoplasma was regularly monitored with the kit “Mycoplasma Stain”.

### 2.3. Cytotoxicity Assay

The cytotoxicity assays were carried out following the methods reported by Ruiz et al. [24] with modifications. SH-SY5Y cells were cultured at a density of 30,000 cells per well in 96-well plates. Following 48 h, the culture medium was refreshed with new medium containing CIT in serial dilutions (from 19.38 up to 310 μM by dilution 1/2). Cells were exposed to the mycotoxin for 24 and 48 h without medium or mycotoxin replenishment.

MTT assay relies on the conversion of a yellow soluble tetrazolium salt to a blue insoluble formazan product by the mitochondrial succinic dehydrogenase enzyme, indicative of viable cell metabolism [24]. For MTT, after 24 and 48 h of exposure to varying concentrations of CIT, the culture medium containing the mycotoxin was replaced with 200 μL of fresh medium and 50 μL of MTT solution (5 mg/mL in PBS) added to each well. Following 3 h incubation at 37 °C in the dark, the formazan was dissolved in DMSO. Absorbance was recorded at 570 nm with a VICTOR2 1420 (Perkin Elmer, Turku, Finland).

The neutral red (NR) assay, which measures NR dye uptake and retention in lysosomes, was conducted by pre-incubating the NR solution overnight at 37 °C and filtering it (0.22 μm filter) to avoid dye crystals [25]. Following 24 and 48 h of exposure to CIT, the medium was changed to the NR solution (200 μL) at a concentration of 50 μg/mL (pre-warmed at 37 °C). The plates were then incubated for 3 h at 37 °C. Afterward, PBS was used to wash the cells, and they were fixed using a formaldehyde–CaCl_2_ solution (0.5% formaldehyde and 1% CaCl_2_ in PBS), followed by extraction with acetic acid–ethanol (1% acetic acid and 50% ethanol in PBS). The plates were shaken for 5 min and then the absorbance was measured at 540 nm with a Multiscan EX ELISA reader (Thermo Scientific, Boston, MA, USA).

For both assays, the values of cell viability were calculated as a percentage of the solvent control (≤1% DMSO). Each exposure time was tested three times in independent experiments (8 replicates per experiment). The IC_50_ values were determined employing SigmaPlot 11 software (Systat Software Inc., GmbH, Erkrath, Germany).

The CIT concentrations used for the following assays (19.375 to 50 µM) are higher than typical dietary exposures but are within the range of concentrations that could occur in extreme cases of contamination.

### 2.4. Reactive Oxygen Species (ROS) Production Assay

Early ROS generation was assessed using H_2_-DCFDA, a fluorescent probe. The ROS generation assay was performed according to Zingales et al. [7]. Specifically, 30,000 cells/well were seeded in a 96-well plate with black walls. After 48 h, the medium was changed, and the cells were incubated for 20 min with 20 μM H_2_-DCFDA (in cell culture medium). Then, the H_2_-DCFDA solution was replaced with 200 μL/well of a medium containing ≤1% DMSO (control) or CIT at concentrations of 19.38, 25, and 38.75 μM. These concentrations were selected based on previous cytotoxicity assays. All tested concentrations were below the IC_50_ values obtained. Fluorescence was measured at excitation and emission wavelengths of 485 and 535 nm, respectively, using a Wallace Victor2 1420 (PerkinElmer, Turku, Finland), with readings taken at intervals up to 120 min. The results are presented as the increase in fluorescence compared to the solvent control. The experiments were conducted independently twice, with 24 replicates each.

In addition, ROS production at 24 h of CIT exposure was also assessed. In this assay, after confluence, the SH-SY5Y cells were exposed to CIT by replacing the culture medium with fresh medium containing CIT at final concentrations of 25, 38.75, and 50 μM. After 24 h of exposure, the supernatant with CIT was removed, and each well received 200 μL of fresh medium with 20 μM H_2_-DCFDA. The plates were incubated at 37 °C for 30 min, in darkness. Fluorescence measurements were determined at excitation and emission wavelengths of 485 and 535 nm, respectively, using a Wallace Victor2 1420 (PerkinElmer, Turku, Finland). The results were divided by the cell viability at each concentration and expressed in comparison to the control. The experiments were conducted independently in duplicate, with 24 replicates each.

### 2.5. Mitochondrial Membrane Potential (MMP) Assay

To study the changes in mitochondrial membrane potential (MMP) in live cells, Rhodamine 123 (Rh123), a non-toxic fluorescent dye which is rapidly absorbed by cells with functioning mitochondria, was used following the method outlined by Liu et al. [26]. SH-SY5Y cells were seeded in 96-well black culture plates (30,000 cells/well). Once the confluence was achieved, they were treated with CIT at concentrations of 25, 38.75, and 50 μM for 24 h. The results were divided by the cell viability at each concentration and expressed as a fluorescence relative to the control (≤1% DMSO). Two independent experiments were conducted to ensure reliability, with 24 replicates each.

### 2.6. Flow Cytometric Analysis of Cell Death (Apoptosis/Necrosis)

Cell death, occurring through necrosis and apoptosis, was assessed using the annexin V-FITC/PI assay by flow cytometry. Specifically, viable cells were Annexin V-FITC-/PI-, early apoptotic cells were Annexin V-FITC+/PI-, late apoptotic cells, which have progressed to the necrotic phase, were Annexin V-FITC+/PI+, and necrotic cells were Annexin V-FITC-/PI+.

For the Annexin V-FITC/PI double staining assay, 700,000 cells were seeded per well in 6-well plates and cultured for 24 h. They were then treated with CIT at concentrations of 25, 38.75, and 50 μM for 24 h. After trypsinization, the cells were resuspended in Hepes-Ca^2+^ buffer (360 μL). Following 30 min incubation at 4 °C in darkness, 10,000 events were acquired and analyzed by a BD LSRFortessa flow cytometer (BD Biosciences, Franklin Lakes, NJ, USA). Quadrant statistics were used to determine the percentages of each cell population. The experiment was performed three independent times.

### 2.7. Cell Cycle Analysis by Flow Cytometry

To analyze the cell cycle, Vindelov’s Propidium Iodide (PI) staining solution was utilized, adhering to the protocol described by Juan-Garcia et al. [27]. PI (a fluorescent dye) binds to double-stranded DNA, allowing for the precise measurement of cellular DNA content via flow cytometry.

In brief, SH-SY5Y cells were seeded at a density of 700,000 per well in 6-well plates and exposed to CIT at concentrations of 25, 38.75, and 50 µM for 24 h. Subsequently, the cells were treated with trypsin and then kept on ice for 30 min in 860 μL of fresh medium with staining solution. The staining solution was prepared using 40 μg/mL RNase, 0.1% Triton X-100, 10 mM Tris, 50 μg/mL PI, and 10 mM NaCl in PBS. Then, 3 independent experiments were conducted, and 20,000 cells were analyzed from each sample using a BD LSRFortessa flow cytometer (BD Biosciences, Franklin Lakes, NJ, USA).

### 2.8. Assessment of Activation of Bcl2 and Bax by Western Blot

For the Western blot assay, proteins were obtained from SH-SY5Y cells after CIT exposure. The cells were lysed and the proteins were extracted in 200 µL of radioimmunoprecipitation assay buffer (RIPA) with inhibitor cocktail of proteases and phosphatases (Santa Cruz Biotechnology, Santa Cruz, CA, USA). Sample components were centrifuged at 4 °C and 12,000× *g* for 15 min to collect cellular proteins in the supernatants and they were quantified using Bradford assay (BioRad Laboratories, Madrid, Spain) with Cydex modification according to Rabilloud et al. [28]. Equal amounts of proteins in the samples were separated by 7.5% sodium dodecyl sulfate-polyacrylamide gel electrophoresis (SDS-PAGE) and transferred to a polyvinylidenedifluoride (PVDF) membrane (BioRad Laboratories, Madrid, Spain). Membranes were blocked in 5% (*w*/*v*) non-fat milk in buffer TBS-Tween for 1 h at room temperature and then the membrane was incubated with rabbit anti-Bcl2 1:1000 (12789-1-AP, Proteintech (Rosemont, IL, USA)), mouse anti-Bax 1:1000 (60267-1lg, Proteintech (Rosemont, IL, USA)), and rabbit anti-β actin 1:1000 (#4970, Cell Signaling technology (Danvers, MA, USA)) in blocking buffer at 4 °C overnight. Then, membranes with blots were washed three times with TBS-Tween buffer, and were blocked and incubated with horseradish peroxidase (HRP) conjugated-secondary antibodies (1:5000) for 2 h at room temperature. Protein bands were detected with the enhanced chemiluminescence (ECL) method (ThermoFisher Scientific, Cambridge, MA, USA) and quantified with ImageJ software (NIH, Bethesda, MD, USA).

### 2.9. Statistical Analysis

Statistical analyses were performed using GraphPad Prism 8 (GraphPad Software, San Diego, CA, USA). Data are presented as the mean ± SEM from two or three independent experiments. The statistical significance was assessed using Student's *t*-test for paired samples. For multiple comparisons, one-way ANOVA was applied, followed by Tukey’s HSD post hoc test. A *p*-value of ≤0.05 was considered statistically significant.

## 3. Results

### 3.1. Cytotoxicity Tests

The cytotoxic effects of CIT on SH-SY5Y cells were studied using MTT and NR assays. The IC_50_ values obtained by both methods are shown in Table 1. Results obtained by the MTT assay described that the viability of cells exposed to CIT decreased significantly in a concentration-dependent manner (Supplementary Data), but there were no differences between the two exposure times tested (Table 1).

Similarly, in the NR assay, exposure to CIT in SH-SY5Y resulted in a concentration-dependent decrease in cell viability at 24 and 48 h of exposure (Supplementary Data). However, according to the IC_50_ values obtained by NR assay, a higher cytotoxic effect was observed after 48 h of exposure compared to MTT assay (Table 1).

The MTT assay results obtained in our study are very similar to the findings of Klarić et al. [29]. These authors determined cell viability of CIT in PK15 cells after 24 h of exposure and observed an IC_50_ value of 73.5 µM by the MTT assay and 75.4 µM CIT in the trypan blue assay. Additionally, the results obtained in our study for the NR assay are very similar to the value reported by Föllmann et al. [30]. These authors found that the IC_50_ value for CIT after 24 h exposure in V79 cells was 70 µM, and the IC_50_ value at 48 h of exposure was 53 µM. The value obtained at 48 h is highly similar to the one described in our study (54.7 µM), although the result obtained at 24 h is lower than the one obtained by us (101.0 µM) (Table 2).

These results could be explained because the SH-SY5Y cells are more sensitive to CIT compared to other cell types, as these authors evidenced higher IC_50_ values than us in different cell lines. In this sense, Aydin et al. [31] obtained an IC_50_ value of 116.5 µM in a mouse Sertoli cell line after 24 h of exposure. In addition, Gayathri et al. [32] obtained an IC_50_ of 155 µM by MTT assay on HepG2 cells treated with CIT for 24 h (Table 2). The concentrations for the subsequent tests were chosen based on the outcomes of the cytotoxicity tests. The CIT concentrations selected for further experiments were 19.375, 25, 38.75, and 50 µM CIT, depending on the experiment.

**Table 2:** Cytotoxicity of CIT in different cell lines and at different exposure times.

Cell Line	Assay	Exposure Time	IC_50_ (µM)	Reference
TM4	MTT	24 h	116.5	Aydin et al., 2019 [31]
		48 h	28.79	
		72 h	28	
HepG2	MTT	24 h	96.16	Sharath Babu et al., 2017a [33]
C2C12	MTT	24 h	102.04	Sharath Babu et al., 2017b [34]
HepG2	MTT	24 h	155	Gayathri et al., 2015 [32]
V79	Neutral Red	24 h	70	Föllmann et al., 2014 [30]
		48 h	62	
Hep3B	MTT	24 h	124	Anninou et al., 2014 [35]
		48 h	77	
PK15	MTT	24 h	73.5	Klaric et al., 2011 [29]
	Trypan Blue	24 h	75.4	

### 3.2. ROS Production

#### 3.2.1. Early ROS Production

**Figure 1:**
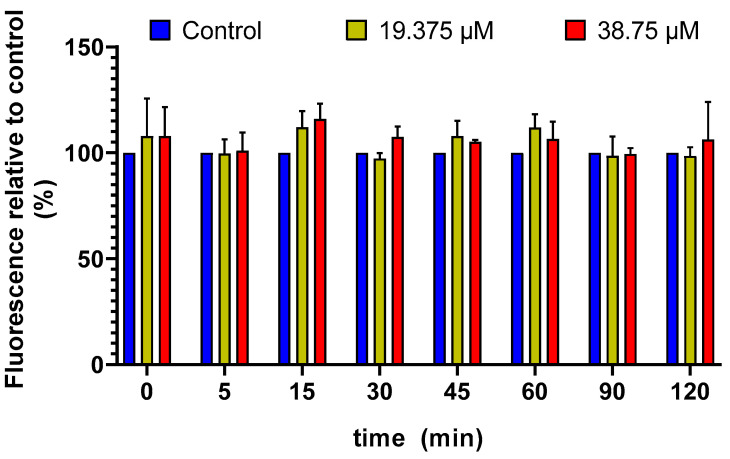
Fluorescence caused by ROS measured in SH-SY5Y cells after exposure to CIT at concentrations of 19.375 and 38.75 µM for 120 min. Results are expressed as mean ± SEM of two independent experiments.

The results obtained measuring for 120 min early ROS production after the addition of CIT to SH-SY5Y cells are shown in Figure 1. ROS production was determined using the H_2_-DCFDA assay. As shown in Figure 1, CIT did not induce ROS generation at the tested concentrations. The levels of ROS production remained similar to the control group during the first 120 min of CIT exposure. According to Vanacloig-Pedros et al. [36], in yeast, the activation of antioxidant genes is part of the cell’s defense mechanism against the oxidative stress caused by CIT. This response is mediated through oxidative stress-responsive transcription factors such as Yap1 and Skn7. Similar mechanisms could explain the lack of elevated ROS levels in other organisms such as mammals. These findings align with the results reported by Mitchell et al. [20], who studied early ROS production in SH-SY5Y cells treated with CIT at concentrations of 15, 30, and 60 µM. They concluded that CIT does not induce toxicity through ROS production as a mechanism of action. Similarly, this finding is in line with previous studies conducted with other mycotoxins, such as sterigmatocystin, where no increase in ROS production was observed within the first 2 h of exposure [7]. Comparable results were obtained by Agahi et al. [37], who described that concentrations below 2.5 μM of α-zearalenol did not generate ROS production during the initial 120 min in SH-SY5Y cells. Finally, Taroncher et al. [38] also reported that exposure to patulin (2.5–10 nM), deoxynivalenol (DON) (25–100 nM), and T-2 toxin (8.5–34 nM) did not increase early ROS production within the first 120 min in HepG2 cells.

#### 3.2.2. ROS Production at 24 h

**Figure 2:**
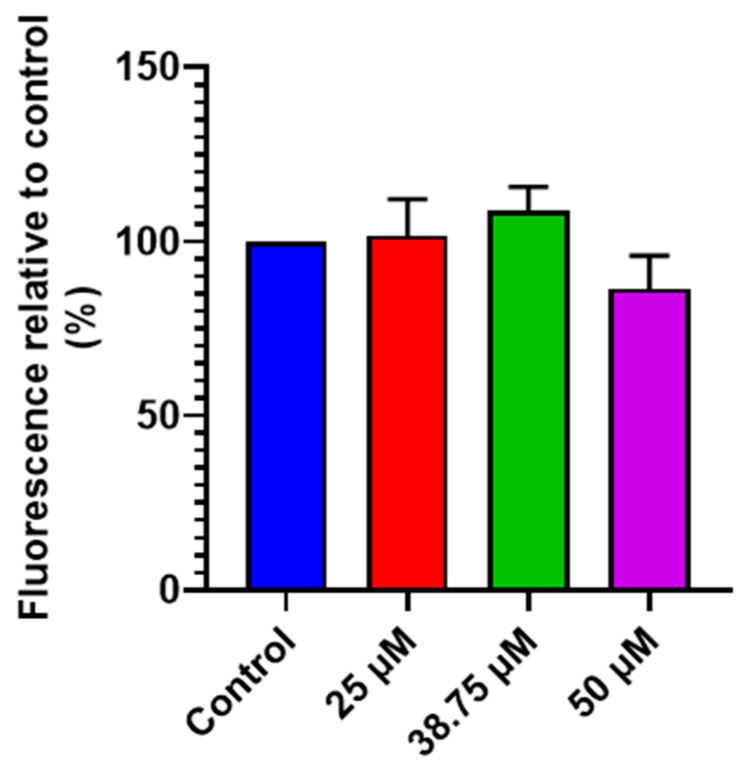
Fluorescence caused by ROS measured in SH-SY5Y cells exposed to CIT (25, 38.75, and 50 μM) for 24 h. Data are expressed as mean ± SEM of two independent experiments.

After analyzing the early production of ROS, a study was conducted to determine ROS generation after 24 h of CIT exposure in SH-SY5Y cells. For this purpose, CIT was added to SH-SY5Y cells at concentrations of 25, 38.75, and 50 μM and ROS production was measured using the H_2_-DCFDA probe after 24 h of CIT exposure in SH-SY5Y cells. The obtained results are shown in Figure 2. As can be observed, there was no significant ROS generation in SH-SY5Y cells between any of the CIT concentrations tested and the control.

Despite the fact that many mycotoxins induce cellular oxidative stress by increasing ROS production, there are also some studies on different mycotoxins that show no increase in ROS generation at 24 h of exposure. In this regard, Cano-Sancho et al. [39] observed that the production of ROS did not increase in Caco-2 cells following exposure to DON for 6, 24, and up to 48 h, concluding that the cytotoxicity of this mycotoxin was not related to ROS production. In addition, according to the findings of Galvano et al. [40,41], it was observed that fumonisin B1 (FB1) did not induce an increase in ROS production in rat astrocytes at 48, 72, and 144 h nor in human fibroblasts at 48 and 72 h. Finally, Janik-Karpinska et al. [42] did not observe significant changes in ROS levels after exposing Hs68 cells to the mycotoxin T-2 (0.001–100 µM) for 24 and 48 h.

### 3.3. Evaluation of Changes on Mitochondrial Membrane Potential (MMP)

**Figure 3:**
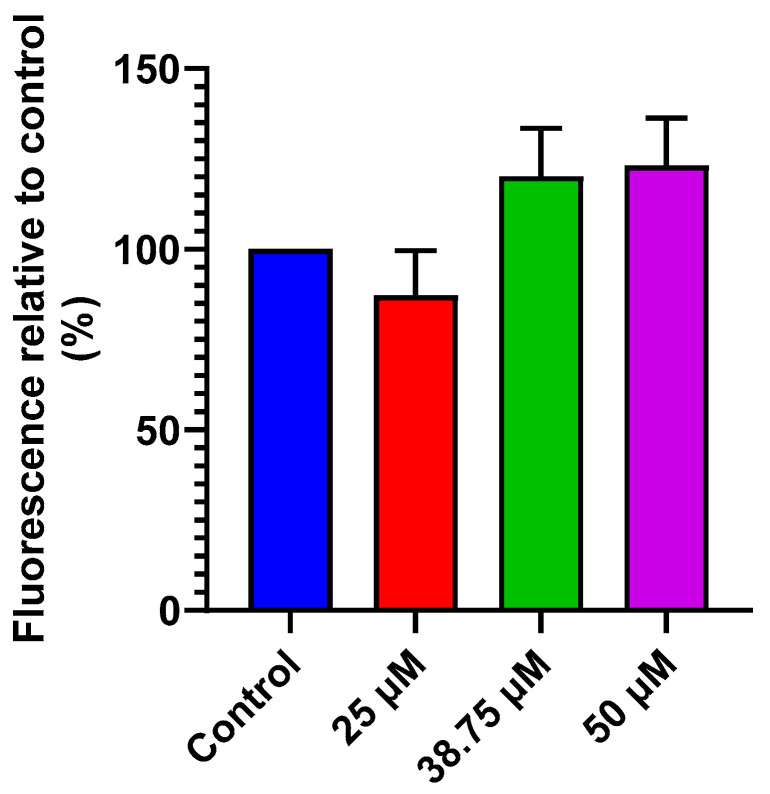
Effect of CIT (25, 38.75, and 50 μM) at 24 h of exposure on Rhodamine 123 (Rh123) assay used to measure mitochondrial membrane potential (MMP). Results are presented as mean ± SEM from two independent experiments.

Mitochondrial function was assessed in SH-SY5Y cells exposed to several concentrations of CIT (25, 38.75, and 50 μM) for 24 h. The changes in MMP were analyzed using the Rh123 probe. The results showed no significant MMP alteration when SH-SY5Y cells were incubated with increasing concentrations of CIT (Figure 3). These findings suggest that the cytotoxic effects induced by CIT may not be directly associated with MMP alteration. However, further investigation is needed to better understand the impact of CIT exposure on mitochondrial functional activities in SH-SY5Y cells.

Our findings are similar with those obtained in the literature in this field. For instance, Pérez-Fuentes et al. [43] reported no significant changes in MMP when SH-SY5Y cells were treated with 0.1 to 30 µM FB1 for 6 and 24 h and DON for 6 h. However, conversely to our results, other mycotoxins induce mitochondrial dysfunction. Pang et al. [44] observed that 5 and 10 ng/mL of T-2 toxin reduced MMP to 60.7% and 41.5%, respectively, in comparison to the control cells. Furthermore, FB1 at 20 and 40 μg/mL was found to cause mitochondrial damage and a loss of MMP in IPEC-J2 cells [45]. Similarly, Zhao et al. detected a decrease in MMP in Het-1A cells exposed to OTA for 24 h [46].

As can be observed, when comparing the results obtained by Pérez-Fuentes et al. and Pang et al. regarding FB1, it becomes evident that there are notable differences in MMP effects produced depending on the cell line used in each assay.

### 3.4. Apoptosis Analysis

**Figure 4:**
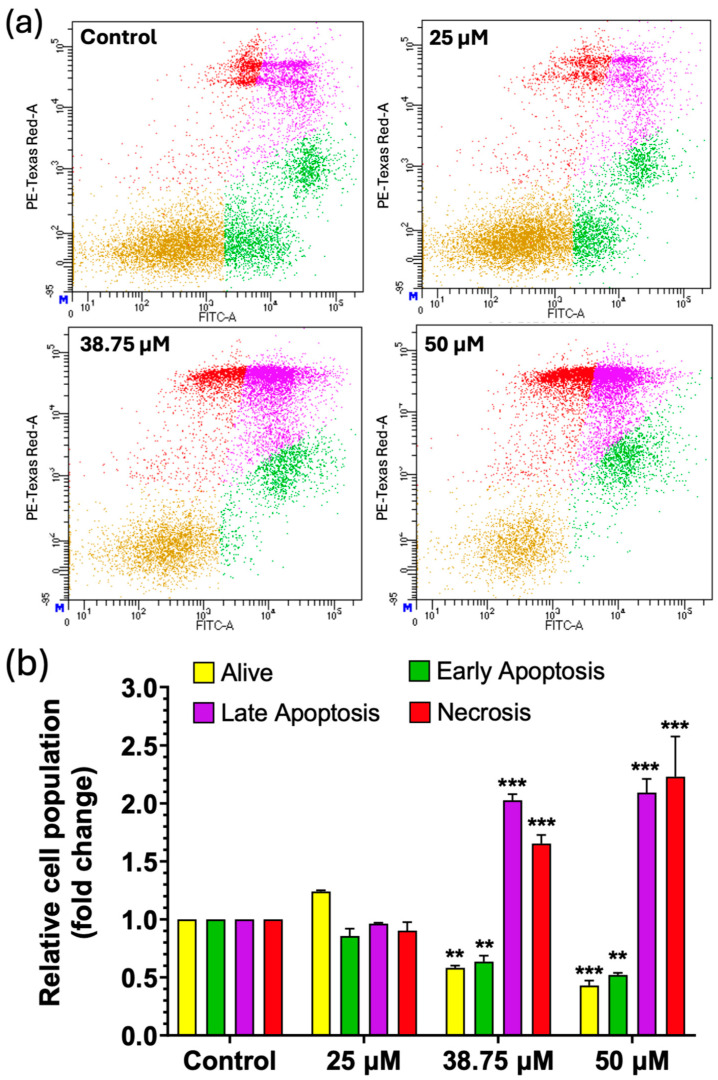
(**a**) Dot plot of control SH-SY5Y cells (≤1% DMSO) and SH-SY5Y cells exposed to 25, 38.75, and 50 μM for 24 h. Yellow: Alive cells (Annexin V-FITC-/PI-); green: early apoptotic cells (Annexin V-FITC+/PI-); purple: late apoptotic cells (Annexin V-FITC+/PI+); and red: necrotic cells (Annexin V-FITC-/PI+). (**b**) Analysis of apoptosis and necrosis processes induced in SH-SY5Y cells exposed to CIT at 25, 38.75, and 50 μM for 24 h. Results are expressed as mean ± SEM of three separate experiments. ** *p* < 0.01 vs. control; *** *p* < 0.001 vs. control.

Since there was a decrease in cell viability depending on the CIT concentration tested, it was essential to study the mechanisms triggering cell death. For this purpose, the apoptotic effect was measured in SH-SY5Y cells. Figure 4 presents the distribution of viable, early apoptotic, late apoptotic, and necrotic cells after CIT exposure (25, 38.75, and 50 μM) in SH-SY5Y cells for 24 h.

Results in Figure 4 showed that for the cells exposed to CIT, the late apoptotic and necrotic cell population increased compared to the control cells. Regarding late apoptotic cells, this increase was similar for 38.75 μM CIT (2.026 ± 0.052 folds) and for 50 μM CIT (2.093 ± 0.117 folds), against the control. Concerning necrosis, there was an increase in the concentration-dependent manner of necrotic cells exposed to CIT, being 1.653 ± 0.074 and 2.229 ± 0.348 times higher for 38.75 and 50 μM CIT, respectively, compared to the control.

The proportion of early apoptotic cells after exposure to CIT for 24 h showed a slight decrease at concentrations of 38.75 and 50 μM, likely due to the extended exposure time (24 h) and an increase in the late apoptotic cell population. Finally, the proportion of living cells decreased as expected for 38.75 and 50 μM, while there was a not significant increase in this population at 25 μM, probably explained by the hormesis effect.

No significant differences were observed at 25 μM, the lowest concentration tested, for any of the studied processes.

The results obtained in our study are similar to those obtained in the literature. Salah et al. found that 150 µM CIT induced apoptosis in HCT116 cells by triggering endoplasmic reticulum stress through the activation of the mitochondrial pathway after 24 h of exposure [47]. This finding was also observed by Yu et al. in their study on HL-60 cells exposed to CIT (25, 50, and 100 μM) for 24 h [16]. Similarly, Sharath Babu et al. [34] investigated the effects of CIT on C2C12 cells and concluded that this mycotoxin was able to induce apoptosis.

### 3.5. The Study of the Cell Cycle

**Figure 5:**
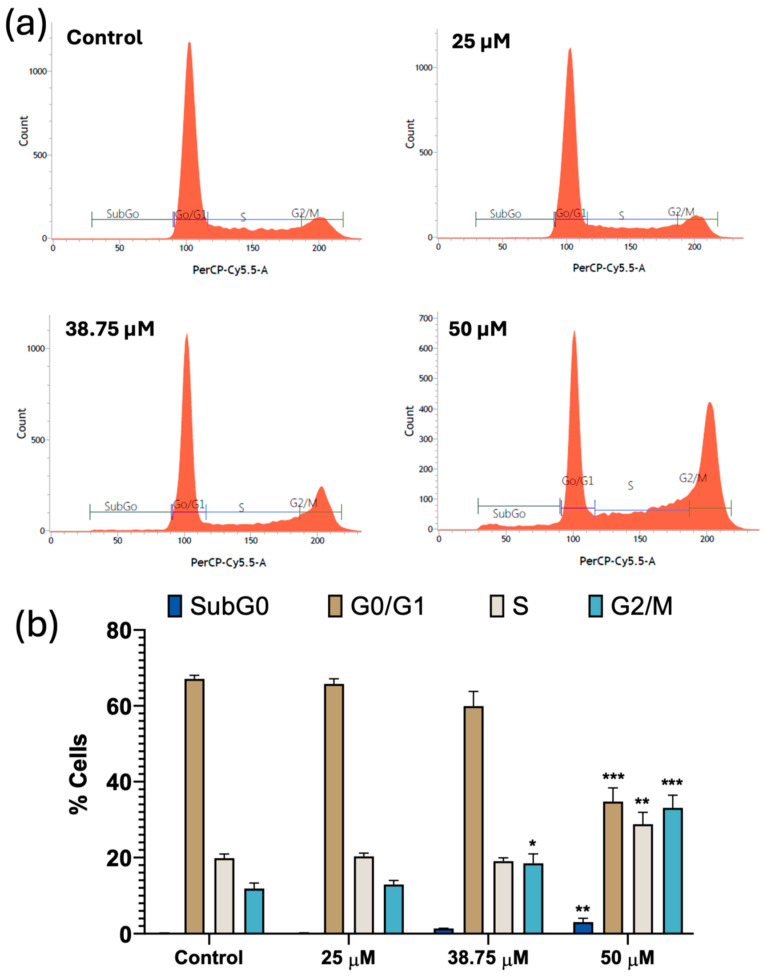
(**a**) Cell cycle distribution analysis of SH-SY5Y cells treated with CIT at concentrations of 25, 38.75, and 50 μM for 24 h. Data are shown as mean ± SEM from three independent experiments. Statistical significance is indicated as follows: * *p* < 0.05 vs. control; ** *p* < 0.01 vs. control; *** *p* < 0.001 vs. control for each cell cycle phase. (**b**) Cell cycle histograms for control SH-SY5Y cells (≤1% DMSO) and SH-SY5Y cells exposed to 25, 38.75, and 50 μM CIT for 24 h.

To study the impact of CIT (25, 38.75, and 50 µM) on the cell cycle, a flow cytometry analysis was carried out on SH-SY5Y cells incubated with Vindelov’s PI staining reagent after 24 h of treatment (Figure 5). The highest CIT concentration (50 µM) showed statistically significant differences compared to the control group in all cell cycle phases. There was a significant reduction in the percentage of cells in the G0/G1 phase, while the percentages of cells in the S and G2/M phases increased. Specifically, the percentage of SH-SY5Y cells in the G0/G1 phase dropped from 67.10 ± 0.58% (control) to 34.77 ± 2.08% (50 µM CIT-treated). The S phase showed an increase, reaching 28.80 ± 1.80%, which is higher than the control condition (19.83 ± 0.64%). Similarly, the G2/M phase exhibited an increase, with the proportion of cells rising to 33.10 ± 1.94%, compared to the control (11.83 ± 0.84%). Moreover, the subG0 phase also experienced a significant increase at the highest concentration, reaching 3.07 ± 0.70%, higher than the control value (0.15 ± 0.04%). Additionally, at 38.75 µM CIT, there was a significant increase in the G2/M phase (18.47 ± 1.44%) compared to the control. The lowest concentration studied, 25 µM CIT, did not exhibit any significant changes in any phase in comparison to the control cells.

Our results are similar to those obtained by Föllmann et al. [30]. They observed that a concentration equal or above 50 µM CIT induced G2/M phase arrest in V79 cells. In addition, their study also reported an increase in the SubG0 population (apoptotic cells), which corresponds with the results observed in our study. Similarly, Chang et al. [14] identified G2/M phase arrest in HEK293 cells following exposure to 50, 75, and 100 μM CIT for 24 h. Specifically, they noted a G2/M phase population of 29.0 ± 3.2% at a concentration of 50 μM, which closely resembles our findings of 33.10 ± 1.94%.

Furthermore, Kumar et al. conducted an in vivo study to determine the effects of CIT topically applied in mice [13]. When mice were exposed to CIT (from 24 to 72 h), there was a notable increase in the cell population in the G0/G1 phase (from 45% to 71%) and there was a decrease in the S phase (from 44% to 59%), in comparison to the control group. Nevertheless, there were no significant changes observed in the G2/M phase following CIT exposure for 12 to 24 h, but an increase in the G2/M phase was evidenced after 48 and 72 h of exposure (an increase of 65% and 56% more, respectively, compared to the control).

### 3.6. Protein Expression Analysis by Western Blot

To confirm the presence of apoptosis generated by exposure to CIT in SH-SY5Y cells, the protein expression of Bax and Bcl-2 (apoptosis related markers) was evaluated by Western blot. Since results in cell cycle and apoptosis assays (measured by flow cytometry) showed significant differences after SH-SY5Y cells were exposed at concentrations of 38.75 μM and 50 μM CIT, we decided to use the same concentrations for this assay. In addition, the 25 µM concentration was also maintained in order to study the process when cells were exposed to a low concentration, which did not appear to induce apoptosis in the flow cytometry study.

**Figure 6:**
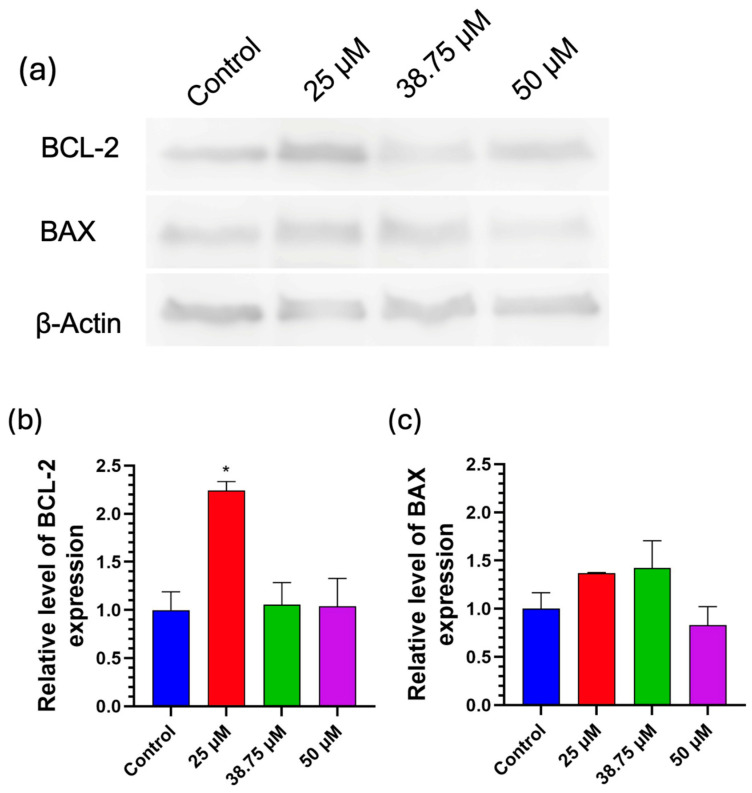
Expression of Bax and Bcl-2 in SH-SY5Y cells after CIT exposure. (**a**) Western blot against Bcl-2 and Bax proteins with loading control protein β-actin in SH-SY5Y cells after exposure to 25, 38.75, and 50 μM CIT for 24 h. (**b**,**c**) Densitometric analysis plot for Bcl-2 and Bax against loading control β-actin. Results are expressed as mean ± SEM of three independent experiments. * = *p* < 0.05 vs. control for each condition.

The Bcl-2 family of proteins plays a central role in regulating apoptosis. This family can be divided into two classes: the anti-apoptotic members, such as Bcl-2, which suppress cell death, and the pro-apoptotic members, such as Bax. The balance between Bcl-2 and Bax is critical in the apoptotic process: Bcl-2 acts to inhibit apoptosis by preventing the release of cytochrome c from the mitochondria, while Bax promotes apoptosis by facilitating this release [48,49]. The results for protein expression are shown in Figure 6. As can be observed, at 25 μM CIT, Bcl-2 expression showed a significant increase compared to the control condition. This may explain the inhibition of the apoptosis process, and an increase in cell viability, as previously observed in the apoptosis/necrosis assay performed by flow cytometry (Figure 4). However, at 38.75 and 50 μM, Bcl-2 expression remains similar to the control; this effect could be because the process of apoptosis is at a more advanced stage. This effect is also in agreement with the effect previously observed in the late apoptosis phase, where according to Figure 4, the cells were mainly in late apoptosis. With respect to Bax expression, no significant differences with the control were observed. Late apoptosis is characterized by the loss of plasma membrane integrity and could be related to an eventual transition to necrosis. The overlap between these processes complicates the quantification of Bax in our model, as cells undergoing late apoptosis may exhibit altered Bax levels by cellular stress and damage [50]. Moreover, in the context of SH-SY5Y cells, studies have shown that various agents can alter the expression of these proteins. For instance, it was reported that certain treatments can lead to a decrease in Bcl-2 levels while not significantly affecting Bax levels, thereby tipping the balance toward apoptosis [51].

On the other hand, an increase in the Bax/Bcl-2 ratio after CIT exposure was previously observed by other authors. In this sense, Wu et al. demonstrated apoptosis with higher doses than those used in our study (from 50 up to 450 µM CIT) [52]. Finally, other authors described a decrease in Bcl-2 and/or an increase in Bax protein expression in in vivo models. Wu et al. [53] observed an increase in the Bax/Bcl-2 ratio in testicle cells from mice treated with 5 and 20 mg CIT/kg body weight. Moreover, Kumar et al. observed a reduction in Bcl-2 and an increase in Bax protein expression after the topical application of CIT (50 µg) in mice and an exposure time from 24 up to 72 h [13]. Similar results were also observed in mouse liver by Wu et al. [15].

## 4. Conclusions

In summary, our findings provide additional information on the cytotoxicity of CIT in SH-SY5Y cells and establish a basis for future research into the full mechanism of action of this mycotoxin. Our findings indicate that CIT induces cell cycle arrest at the G2/M and S phases in SH-SY5Y cells. Moreover, we have identified new aspects of the cell death pathways activated by CIT in these cells, showing an increase in late apoptosis, corroborated by an initial increase in Bcl2 protein expression at the lowest concentration tested followed by a reduction that was dependent on CIT concentration at higher concentrations. However, further studies are needed on shorter exposure times than 24 h to better understand the mechanisms of action by which CIT produces cytotoxicity in SH-SY5Y cells. SH-SY5Y neuroblastoma cells share key damage and survival pathways with other human cells, making them a valuable model for studying this mycotoxin’s cytotoxic mechanisms. Their use is particularly relevant for confirming and extending findings from other cell types, providing additional insights into the toxic effects. In conclusion, these findings will help us to develop effective strategies to prevent CIT initiating molecular and cellular adverse events. Moreover, they facilitate the pathway to regulatory acceptance of existing New Approach Methodologies (NAMs) for CIT cytotoxicity that are both animal-free and human-relevant.

## Figures and Tables

**Table 1:** IC_50_ values for CIT in SH-SY5Y cells, determined using MTT and NR assays after 24 and 48 h of exposure. Results are presented as mean ± SEM from three separate experiments.

IC_50_ Value (µM)	24 h	48 h
MTT	77.1 ± 10.1 µM	74.7 ± 9.6 µM
NR	101.0 ± 20.3 µM	54.7 ± 7.4 µM

## Data Availability

The original contributions presented in this study are included in the article/Supplementary Material. Further inquiries can be directed to the corresponding author.

## Supplementary Materials

The following supporting information can be downloaded at: https://www.mdpi.com/article/10.3390/foods14030356/s1, Figure S1. Dose-dependent cytotoxicity values of citrinin (CIT) on SH-SY5Y after 24 h (a, c) and 48 h (b, d) of exposure, measured by MTT (a, b) and Neutral Red (NR) (c, d) assays. Results are expressed as mean ± SEM of three separate experiments.
