# Supplementary material for: Citrinin-Induced Cellular Damage: Insights from SH-SY5Y Cell Line Studies
Source: Foods. 2025 Jan 22;14(3):356. doi: 10.3390/foods14030356 (PMC11817462; doi:10.3390/foods14030356)
Supplement: Supplementary file 1 [file foods-14-00356-s001.zip › foods-3421038-supplementary.pdf]

foods

MDPI

Article

Citrinin-Induced Cellular Damage: Insights from SH-SY5Y Cell Line Studies

Francisco J. Martí-Quijal [1,2], Felipe Franco-Campos [2,*], Francisco J. Barba [1] and María-José Ruiz [2]

[1] Research Group in Innovative Technologies for Sustainable Food (ALISOST), Nutrition, Food Science and Toxicology Department, Faculty of Pharmacy, Universitat de València, Avda. Vicent Andrés Estellés, s/n, 46100 Burjassot, València, Spain; francisco.j.marti@uv.es (F.J.M.-Q.); francisco.barba@uv.es (F.J.B.)

[2] Research Group in Alternative Methods for Determining Toxics Effects and Risk Assessment of Contaminants and Mixtures (RiskTox), Laboratory of Food Chemistry and Toxicology, Faculty of Pharmacy and Food Science, University of Valencia, Av. Vicent Andrés Estellés, s/n, 46100 Burjassot, València, Spain; m.jose.ruiz@uv.es

* Correspondence: felipe.franco@uv.es

Abstract: Citrinin (CIT), a mycotoxin commonly found in cereals, is produced by fungi from the *Aspergillus*, *Penicillium*, and *Monascus* genera. While its nephrotoxic effects are well studied, its impact on neurons is less understood. This study investigates CIT-induced toxicity in human neuroblastoma cells (SH-SY5Y). The IC_{50} values for cells treated with CIT were 77.1 μM at 24 h and 74.7 μM at 48 h using MTT assay, and 101.0 μM at 24 h and 54.7 μM at 48 h using neutral red assay. CIT exposure caused G2/M phase arrest, with cells in this phase increasing from 11.83% (control) to 33.10% at 50 μM CIT. At 50 μM, the percentage of cells in the S phase also increased, which may suggest that cellular stress pathways were activated. Moreover, an increase in late apoptosis process was noted in cells exposed to CIT for 24 h, particularly at the highest concentrations (38.75 and 50 μM). Western blot analysis confirmed a rapid change in the anti-apoptotic protein Bcl-2, but no significant changes in Bax. In conclusion, CIT induces apoptosis and cell cycle arrest in SH-SY5Y cells. However, further transcriptomic studies in specific proteins involved in different pathways described in this work are needed to gain a comprehensive understanding of the specific mechanisms underlying CIT's toxicity in SH-SY5Y cells.

Keywords: citrinin; cell cycle; SH-SY5Y; ROS; mitochondrial membrane potential

Academic Editor: Silvia Pichardo

Received: 26 December 2024
Revised: 14 January 2025
Accepted: 16 January 2025
Published: 22 January 2025

Citation: Martí-Quijal, F.J.; Franco-Campos, F.; Barba, F.J.; Ruiz, M.-J. Citrinin-Induced Cellular Damage: Insights from SH-SY5Y Cell Line Studies. *Foods* **2025**, *14*, 356. https://doi.org/10.3390/foods14030356

Copyright: © 2025 by the authors. Licensee MDPI, Basel, Switzerland. This article is an open access article distributed under the terms and conditions of the Creative Commons Attribution (CC BY) license (https://creativecommons.org/licenses/by/4.0/).

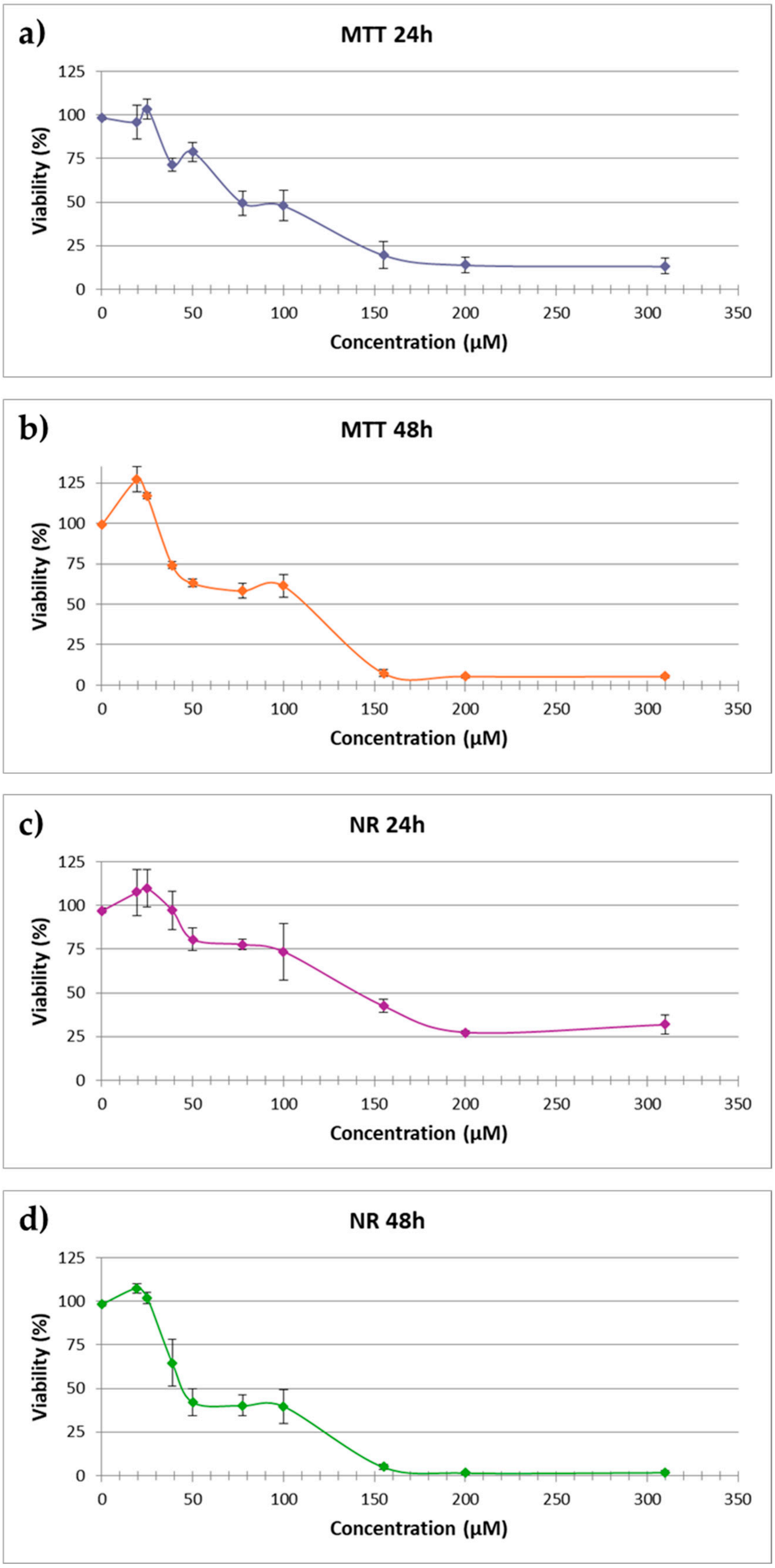

Figure S1. Dose-dependent cytotoxicity values of citrinin (CIT) on SH-SY5Y after 24 h (a, c) and 48 h (b, d) of exposure, measured by MTT (a, b) and Neutral Red (NR) (c, d) assays. Results are expressed as mean ± SEM of three separate experiments.
